# Cancer control in developing countries: using health data and health services research to measure and improve access, quality and efficiency

**DOI:** 10.1186/1472-698X-10-24

**Published:** 2010-10-13

**Authors:** Timothy P Hanna, Alfred CT Kangolle

**Affiliations:** 1 Cancer Centre of Southeastern Ontario 25 King Street West, Kingston, ON, K7L 5P9, Canada; 2 Ocean Road Cancer Institute P.O. Box 38158 Dar Es Salaam, Tanzania

## Abstract

**Background:**

Cancer is a rapidly increasing problem in developing countries. Access, quality and efficiency of cancer services in developing countries must be understood to advance effective cancer control programs. Health services research can provide insights into these areas.

**Discussion:**

This article provides an overview of oncology health services in developing countries. We use selected examples from peer-reviewed literature in health services research and relevant publicly available documents. In spite of significant limitations in the available data, it is clear there are substantial barriers to access to cancer control in developing countries. This includes prevention, early detection, diagnosis/treatment and palliation. There are also substantial limitations in the quality of cancer control and a great need to improve economic efficiency. We describe how the application of health data may assist in optimizing (1) Structure: strengthening planning, collaboration, transparency, research development, education and capacity building. (2) Process: enabling follow-up, knowledge translation, patient safety and quality assurance. (3) Outcome: facilitating evaluation, monitoring and improvement of national cancer control efforts. There is currently limited data and capacity to use this data in developing countries for these purposes.

**Summary:**

There is an urgent need to improve health services for cancer control in developing countries. Current resources and much-needed investments must be optimally managed. To achieve this, we would recommend investment in four key priorities: (1) Capacity building in oncology health services research, policy and planning relevant to developing countries. (2) Development of high-quality health data sources. (3) More oncology-related economic evaluations in developing countries. (4) Exploration of high-quality models of cancer control in developing countries. Meeting these needs will require national, regional and international collaboration as well as political leadership. Horizontal integration with programs for other diseases will be important.

## Background

2005 estimates suggest 13% of all deaths globally are from cancer [1]. Of the estimated 7.6 million cancer deaths worldwide in 2007, 62% were in developing countries [2]. Given the population boom and aging of the population in developing countries, Parkin et al have projected a 75% increase in cancer incidence in the developing world between the year 2000 and 2020 [3]. In order to prevent and manage this change in cancer incidence, significant organization and infrastructure is required to prevent, screen, treat and palliate cancer. As a result, the implications to health services delivery in developing countries are enormous.

Health services research largely concerns itself with the factors influencing the need for health services, access to these services, quality and economic efficiency, in addition to the health outcomes of the individuals and populations involved [4]. Other health service elements of great importance to developing countries are equity and sustainability. The purpose of this article is to provide an overview of what is known about oncology health services in developing countries and to highlight data needs and current gaps in knowledge. We will focus on elements of access, quality and efficiency. We will then illustrate how filling current gaps in knowledge can facilitate optimization of much-needed cancer-related investment in health care systems and national cancer control strategies.

## Discussion

### Describing Quality of Cancer Control in Developing Countries

We consider the four principal approaches of cancer control to be cancer prevention, early detection, diagnosis/treatment and palliation, as described by the World Health Organization [5]. We will describe what is known about the quality of oncology services for cancer control in developing countries in terms of Donabedian's framework [6]. Donabedian describes quality assessment in terms of structure, process and outcome [6]. **Structure **refers to the attributes of the setting where care is delivered. This includes material resources, human resources and organizational structure. To assess structure, one must consider how well the health care system interfaces with society and individuals in society [7]. For example, a current structural problem for developing countries is a medical structure historically designed to manage communicable disease, nutritional deficiencies, child health and maternal health. These health care structures are now faced with the additional burden of chronic diseases such as cancer, cardiovascular disease, diabetes and chronic respiratory disease. These chronic diseases share common risk factors, emphasizing the importance of integration between prevention programs targeting them. **Process **refers to what is actually done while care is provided and received. This includes patient's health care seeking activities, the diagnostic process, treatment recommendation and treatment delivery. **Outcome **refers to the effects of care on the health status of patients and populations. Outcomes may be medical parameters (e.g. survival after cancer treatment), quality of life, patient satisfaction, equity or economic efficiency [7]. Outcomes also include measures such as changes in patient knowledge and individual's behaviour.

### Structure of Oncology Services in Developing Countries

There is a limited but growing body of literature describing the current status of cancer treatment resources, particularly for radiation therapy, in developing countries. The emerging picture demonstrates extreme limitation of human resources, physical resources and equipment. Taking into account the annual incidence of cancer in developing countries, Barton et al found that radiation machine supply was in general inadequate to meet demand [8]. Machine supply in Africa was sufficient to meet an estimated 18% of the need for radiation (155 megavoltage machines available, 842 needed in total). For 12 countries in the Asia-Pacific region with available data (including India and China), machine supply was sufficient to meet an estimated 23% of the current need (1147 available, 4936 needed). Significant shortfalls were also found in Latin America and Eastern Europe. This data highlights just one example of the potential for mismatch between patient need and treatment availability.

Available information suggests there is an overall shortage of health care workers in Low- and Middle-Income Countries, as well as massive disparities between the rich and poor regions of the world. The World Bank defines Low-Income Countries as those with a 2008 gross national income (GNI) per capita of US$975 or less. Lower-Middle-Income and Upper-Middle-Income Countries are defined as those with a GNI per capita of US$976 - $3,855 and US$3,856 - $11,905 respectively [9]. These groupings provide an indication of the range of economic situations among developing countries. For reference, High-Income Countries are those with a GNI per capita of US$11,906 or more. High-Income countries of the world are largely in North America and Europe; other High-Income Countries include Japan and Australia. Europe has 11 times the number of nurses per population compared to South-East Asia and 7 times the number of doctors [10]. North America, on average, has 10 times more nurses per population than countries in South America [11].

Due to limited data in many developing countries, little is known regarding capacity for treatment with chemotherapy and surgery, capacity for cancer prevention, early detection and other services. It is reasonably assumed that human capacity for these activities is extremely limited given the global shortage of human resources for health care in developing countries.

Narcotics such as morphine are irreplaceable for controlling severe pain from cancer and other serious medical conditions. However, global morphine consumption trends provide evidence of gross under-utilization of narcotics for pain control in many Low-Income and Middle-Income Countries [12]. For example, in 2004, Canada reported 64.180 mg morphine consumed per capita, compared to 0.420 mg per capita for Peru, 0.020 mg per capita for Indonesia and 0.001 mg per capita for Mozambique [12].

Publicly available data regarding access to drugs for cancer therapy is limited. A 2001 country-level survey by the World Health Organization found only 22% of African countries reported availability of anti-cancer drugs, 43% in the South East Asia region and 57% in the Americas [13]. This compared to 91% in Europe. As there are significant limitations to access in many developing countries to essential drugs for chronic diseases other than cancer, it is quite likely that access to cancer drugs is at least as significant a problem [14,15]. Additional challenges unique to cancer drugs include the need for extra safety equipment for pharmacy staff preparing them for administration. This equipment can be quite costly. Also, some cancer drug therapies require predictive tests prior to use, for instance, the common need for hormone receptor status prior to hormone therapy for breast cancer. This poses an additional barrier due to issues of cost and quality control.

The exact number of men, women and children dying of cancer without appropriate diagnosis, treatment, early detection or palliative care is unknown. However, available information suggests that the number is probably staggering. The role of social, economic and political factors such as the impact of a limited state presence in health issues or wide-spread poverty in explaining disparities such as these is not always known. A political economy approach that considers the role of these factors warrants careful thought.

### An Illustration: The Situation in Tanzania

Tanzania is a Low-Income Country. In 2008, it had a population of 42.5 million people and an estimated 21,180 new cancers [16,17]. Resources to manage this significant burden of cancer are limited. For example, nation-wide, there is 1 medical oncologist, 4 radiation oncologists, 2 physicists and an estimated 7 pathologists. No surgeon's practice is dedicated solely to oncology. These human resources are extremely limited compared to actual need. For perspective, in Canada, a radiation oncologist would typically see 250 new patients per year. Given at least half of East African cancer patients will need radiation by one conservative estimate, if each Tanzanian radiation oncologist saw 250 new patients per year, this would place the number of radiation oncologists in Tanzania dramatically below the estimated need [8].

Breast and cervix cancer are the most common killers due to cancer among Tanzanian women. They contribute approximately 40-50% of all new cancer cases seen at the Ocean Road Cancer Institute, the only specialized cancer treatment institute in Tanzania. At present, there is no national cancer screening program for cervix, breast or other types of cancer. There are only two radiotherapy machines in the country, even though one estimate suggests a need for 45 [8]. Capacity for cancer screening and staging is limited. There are only three mammography units, an estimated five CT scanners and one MRI nationwide. Palliative services are only available in a few towns and three hospitals with the use of morphine limited by national policies. Finally, there is no formal training program within Tanzania for any discipline of oncology, forcing candidates to travel abroad, often to South Africa or Europe for training.

Fortunately, many agencies are partnering in the development of cancer control capacity in Tanzania. For instance, the International Atomic Energy Agency (IAEA) Program of Action for Cancer Therapy (PACT) has been involved in assessing needs for cancer control, planning, building human capacity and developing physical resources.

### Limitations in Quality and Access to Cancer Services in Developing Countries

In Low- and Middle-Income Countries, access to cancer care is often poor. The availability of trained human resources and physical resources are fundamental problems in cancer control among developing countries. However, other factors also limit access to services in developing countries. Penchansky and Thomas' framework of access to care fittingly describes some of these. In addition to availability, they describe spatial accessibility, acceptability, affordability and accommodation [18]. Mackillop highlights a sixth dimension, awareness [19]. Equitable access to cancer control in developing countries is a prerequisite to equitable quality of care [20].

The dimensions of access described by Penchansky, Thomas and Mackillop must be further characterized on national and regional levels for cancer control efforts in limited-resource countries to be optimally suited to their environments. For instance, although the effect of geography on spatial accessibility to cancer treatments has not been sounded in developing countries, it is probably a significant barrier to access. Even in High-Income Countries, distances as short as dozens of kilometres from the patient's home to the radiation therapy centre may be associated with less use of radiation or fewer daily treatments per patient treated [21,22]. In many poor countries, cancer treatments such as radiation are only available at a few sites nationally and often in just one city [23-25]. This is important to note as for the poor, travel costs may exhaust any financial reserve before the first cancer treatment is offered.

Acceptability of treatment may be limited by values surrounding loss of hair or fertility during chemotherapy or loss of organs due to surgery (e.g. mastectomy for breast cancer). These values have rarely been described or quantified in developing countries [26]. Stigma from cancer, cultural factors, personal beliefs, fatalism or fear of treatment may lead to patient-related delay in treatment [26-29]. It is quite likely that many of these same factors would also prevent patients in developing countries from seeking treatment at all. Acceptability issues may also limit access to screening and prevention. For example, embarrassment or fear of pain may keep young Brazilian women from seeking cervical cancer screening [30].

Public awareness campaigns and government health programs for developing countries have often been largely geared towards communicable diseases [31]. The potential impact of building awareness of principles of cancer treatment, prevention and early detection on access to care in developing countries may be tremendous. For example, in Lahore, Pakistan, only 5% of women surveyed at a tertiary care hospital were aware that screening existed for cervix cancer and only 2.6% had ever received a pap test [32]. Only one third of patients seeking medical attention for cancer at a large cancer clinic in Delhi, India believed that cancer could be cured [33]. The situation may be complicated by modest or limited health care worker training in basic principles of oncology and sometimes even a lack of awareness of the curability of cancer [34,35].

Beyond building awareness among the general public, creating an awareness of the need for cancer services in developing countries among government officials, medical educators, policy makers and key advocates in the public and press is crucial. The health services research community has an important role to play in improving awareness on these fronts, informing communications and targeting messages as effectively as possible. Organizations such as the International Union Against Cancer (UICC), the IAEA, the American Cancer Society and the World Health Organization (WHO) have taken leadership in this area, though a substantial effort is still needed to expand awareness among all parties.

### Process of Oncology Services in Developing Countries

Little is known about both the process and outcome of cancer control efforts in most developing countries despite the importance of this information (see below). Descriptions of patterns of cancer treatment in developing countries are hard to find. For example, there is only one report in the indexed literature examining palliative radiation use in Africa. While there is extensive clinical trial evidence supporting shorter and less costly palliative treatments for bone metastases, this report suggests that 77% of surveyed centers use longer treatments, despite limited resources [36]. Information on the equity of the treatment process in this or other settings is not available in the literature to our knowledge.

An IAEA study of patterns of practice in developing Central and Eastern European countries found variations in use of radiation for lung cancer, including schedules of palliative radiation and approaches for radical treatment. Concurrent chemo-radiation is considered the optimal curative treatment for unresected advanced non-small cell lung cancer [37]. Among the developing countries participating in this study, sequential chemo-radiation (57%) was used far more often than concurrent chemo-radiation (10%) for non-small cell lung cancer, the opposite of reported patterns of practice in the United States [38,39].

A patterns of care study from China found that 92% of radiation for breast cancer was given as post-mastectomy radiation therapy (PMRT), suggesting little use of breast conserving therapy among clinicians surveyed [40]. Though low survey response rates limit interpretation of these results, it appears that the non-standard indication of PMRT for centrally or medially located cancers ≤5 cm in size without lymph node spread is common, as is radiation to the internal mammary lymph nodes which is of unproven benefit.

There is little known regarding patient safety and quality control during cancer treatment in developing countries. Much of the available information comes from the IAEA. They have been involved for years in quality control efforts related to radiation therapy in developing countries as well as various other programs to improve cancer management in developing countries [41,42]. For example, an IAEA audit of radiation machine dosimetry found 16% of audited radiation beams in various developing countries did not meet the +/-5% dose acceptance limit on the first test of radiation dosimetric standards [41]. Doses beyond this +/-5% acceptance limit have been associated with severe and even lethal toxicity [43]. 93% of radiation beams met standards by the second iteration of the audit. These data highlight the importance of technical audit in developing countries, as in all countries. While dosimetry appears to be typically acceptable, a small minority of cases can be identified where patient safety is a concern and changes must be made to local practice.

### Outcomes of Cancer Services in Developing Countries

Available data from developing countries suggests high mortality rates for patients with cancer compared to High-Income Countries [44]. Often five-year survival for Low-Income Countries is less than half that seen in more developed countries [44,45]. The variation in survival may be related to differences in resource availability as well as other dimensions of access and quality of cancer treatment, early detection and prevention [45]. Inequities in survival for groups within individual developing countries have not been explored to our knowledge but are probably substantial.

Outcomes of cancer prevention strategies have been investigated in numerous developing country settings, particularly for cervix cancer screening and also breast cancer early detection and tobacco control [46-51]. These outcome studies suggest many of these strategies are effective. This is relevant as one estimate suggests 1.7 million cancer deaths in Low- and Middle-Income Countries were due to potentially preventable causes in 2001 alone [52].

Little is known about other cancer-related outcomes in developing countries such as toxicity of treatment, quality of life, patient satisfaction, equity and economic efficiency. The lack of information on the economic efficiency of most cancer treatments and many preventive measures is of note. Resources are limited in developing countries and choosing the most cost-effective measures is a very important consideration.

### Information Needs to Improve Cancer Control in Developing Countries

Clearly, investment in health care systems in developing countries is required. For oncology, this includes developing a sustainable supply of trained oncology professionals, expanding the supply of treatment equipment, improving drug supply, physical infrastructure and organizational infrastructure for cancer control. Given limited resources, health services researchers and policy makers must explore how these much-needed investments can be best directed for optimal results. For example, the London Declaration on Cancer Control in Africa endorses national cancer control plans that are 'evidence based, priority driven and resource appropriate'. This will require cancer-related health services research with broad local and international collaborations in addition to long-term investment from all parties [53].

The need for better health monitoring and evaluation data in developing countries is widely recognized. Targets set by the International Union Against Cancer (UICC) World Cancer Declaration 2008 include improved measurement of the global cancer burden and measurement of the impact of cancer control interventions [54]. Notably, this year, eight of the largest global health agencies, including the World Health Organization, Bill and Melinda Gates Foundation and the World Bank reached a consensus on health data [55]. This consensus calls for increased investment in health information, efficiency of this investment, development of a common data architecture, strengthening of performance monitoring and evaluation, increased data use in developing countries and improved data access [55].

Common information sources in developing countries are health surveys, birth and death registration, census data, health facility reporting systems, health surveillance data and health administrative data [55]. These common information sources provide information on many disease types. Given information needs across health sectors, we would emphasize the importance of horizontal integration between disease control initiatives in contrast to solely focusing on vertical stand-alone programs. This offers the promise of strengthening the system-wide structure of health care systems in developing countries.

Population-based cancer registries collect information on the occurrence of different types of cancer in a population. The information they contain may also include national or hospital-based information on cancer treatment and vital status. They are one unique form of cancer data of great importance to developing countries. Cancer registries provide the backbone of information infrastructure needed to perform a wide range of oncology health services research. The population-based information on cancer cases can be linked to other population-based and hospital-based health data sources to provide a rich tapestry of information on structure, process and outcome of cancer prevention, screening, palliation and treatment. Common sources of cancer registry information include hospital inpatient records, radiation treatment records, hospital records, pathology records, haematology lab reports, autopsies, death certificates and screening programme records [56].

There are still many developing countries without a cancer registry of sufficient quality. For instance, the population coverage by cancer registries of sufficient quality for inclusion in the International Agency for Research on Cancer's (IARC) 2007 report on global cancer incidence was 1% for Africa, 4% for Asia, and 4% for South and Central America. This compares to 33% in Europe and 80% in North America [56].

Developing cancer registries and health databases do have their own significant costs. Stansfield et al estimate annual per capita costs of a comprehensive health information system of US$0.53 in Low-Income Countries [57]. This may represent a significant health expenditure for many Low-Income Countries, though there is evidence that this investment can be cost-effective [57]. Low-Income Countries may need to rely initially on basic estimates of cancer burden and health needs when developing their first national cancer control plan. However, it is imperative even for these most limited resource countries to create a timeline and vision for the appropriate development of useful cancer data sources.

### Health Data for Quality Cancer Control in Developing Countries: Structure

The information in cancer registries and related health data serve many irreplaceable functions (Table 1). In terms of improving the structure of cancer control, this information contributes to cancer system planning that fits well to local needs [58,59]. Resource constraints in High-Income Country's cancer systems have given rise to methodologies using health information to estimate the need for cancer services and factors affecting access to these services. These methodologies provide a means for minimizing the dual problems of over-provision (unnecessary care) and under-provision (insufficient care) of cancer control resources. To date, this work has largely focused on radiation therapy [22,60-63]. These methodologies would assist developing countries in ensuring accessible and equitable cancer control. They have not been widely applied to cancer control in developing countries to our knowledge.

**Table 1:** Health Data for Quality Cancer Control in Developing Countries

Quality Assessment Category	Use of Health Data to Improve Quality
Structure	Planning: National cancer control programs that are:
	1. Accessible
	2. Equitable
	3. Flexible and iteratively planned
	4. Sustainable
	5. Scalable
	6. Economically efficient

	Collaboration:
	1. Harmonization of national cancer control efforts between groups
	2. Identifying applicable models from other countries

	Transparency:
	1. Participation: Public involvement in decision making
	2. Advocacy: Calls for greater funding
	3. Accountability: To public and funding agencies

	Research Development:
	1. Defining research priorities
	2. Identifying research subjects

	Education: Improving local medical curricula in oncology

	Capacity building: Local training in health policy, epidemiology and health services research

Process	Follow-up: Ensuring follow-up for cancer screening and treatment

	Knowledge translation:
	1. Identifying targets for knowledge translation initiatives
	2. Evaluation of knowledge translation initiatives

	Patient safety and quality assurance: Audit of technical process

Outcome	Evaluation, monitoring and improvement of national cancer control efforts:
	1. Effectiveness
	2. Accessibility
	3. Equitability
	4. Sustainability
	5. Economic efficiency

Regularly collected health data also allows cancer control planning to be flexible and iterative. Continuous data collection can reveal changes in needs and development of critical health issues [64]. By monitoring for these changes, the health care system can more efficiently change to match these needs. This is an important element for ensuring sustainability of the health care system. Continued data collection also allows for adjustment of the scale of programs to current capacity and need [64].

There are now often many local, national and international health agencies involved in health interventions in any given developing country. Effective collaboration is needed to ensure success. Health data on cancer provides a means of harmonizing the efforts of all groups, most importantly in situations where opinions differ [65]. Collection of national health data can also be helpful in determining how appropriate successful models from other countries would be to national conditions [65].

Collection and evaluation of health information promotes transparency. For instance, this information can allow greater participation of the public and other parties in national and regional advocacy for changes to health policy. It also enables national, regional and international agencies to effectively advocate for needed resources for national cancer control by building a clear picture of local needs [65]. It also allows for accountability of parties involved in provision of cancer control to the public and funding agencies [59].

Cancer registry data and health data are crucial for supporting national and regional research structure. They can help define cancer research priorities, for instance, focusing research on cancers with the greatest burden of disease in the population or exploring the potential cost-effectiveness of a new cancer treatment or cancer control strategy to be studied [66]. Cancer registry data may serve as the basis for epidemiological studies of cancer causation in developing countries or identify patients with cancer for other forms of cancer research. For instance, population-based cancer registries can facilitate studies of access to cancer care.

Cancer registries and their linked data also serve as aids to educating health professionals such as by shaping medical curricula to local problems. They can also serve as an important tool in the local training of individuals in health policy, graduate level epidemiology and health services research. This can ultimately support an evidence-based, research-informed culture of medical practice for participating developing countries. It can also ensure a sustainable supply of workers who can utilize health data in-country to improve the quality of national cancer control.

### Health Data for Quality Cancer Control in Developing Countries: Process

In terms of process, cancer registries and integrated health data systems may facilitate follow-up of individuals for screening or follow-up of cancer patients. They can also assist in understanding the medical decision making process which can help identify opportunities for knowledge translation. For instance, the study of radiation use for bone metastasis in Africa suggests an opportunity to reduce the number of daily radiation treatments per patient. This would increase capacity to treat many more patients while still offering substantial benefit to all, with little added structural costs. Health data can also be used to evaluate promising knowledge translation initiatives.

Health data can be useful in auditing the technical process of cancer control for patient safety and quality control. A high level of technical expertise is required to perform cancer surgery, palliate cancer symptoms effectively, treat with radiation accurately and safely administer chemotherapy. Rates of medical error and factors associated with medical error in cancer treatment in developing countries are largely unknown. A comprehensive review of the literature by the Radiotherapy Safety Team within the World Alliance for Patient Safety found no detailed reports from Africa or Asia on adverse events from radiotherapy [67]. One audit of a new radiotherapy centre in a developing country in Asia found that 52% of audited patients received sub-optimal radiation treatment, potentially leading to compromised cure or palliation or serious treatment toxicity [68]. Inadequate skills and knowledge, lack of a formal quality assurance process or peer review process and heavy workload were identified as barriers to quality. One approach to ameliorating deficiencies is regular treatment audit. This can be enabling for oncology staff and allow targeted continuing medical education [68].

### Health Data for Quality Cancer Control in Developing Countries: Outcome

Collection of health care data allows outcome assessment of cancer control programs. This includes trending changes in an outcome such as cancer incidence or survival over time, or following geographic variations in incidence of a given cancer. Health data can help assess effectiveness, access to care, equitability of structure, process and outcome, sustainability and economic efficiency. In the following section, we will discuss the assessment of effectiveness and economic efficiency as examples. Regarding access and equity, we would briefly mention that methodologies to identify the poor in developing countries do exist, though to our knowledge, they have not been used as part of outcome assessments of cancer control in developing countries [69].

Without health data collection on structure, process and outcome, it will not be possible to effectively evaluate innovative program's effectiveness. For instance, Datta and Rajasekar suggest a three-tier model for provision of radiotherapy services for India [70]. They describe three levels of treatment capacity with smaller centres in the periphery electronically linked to more comprehensively equipped centres. As another example, the role of primary community health care workers in improving cancer control deserves further investigation. Their beneficial roles could include coordinating cancer care, ensuring timely referral, optimizing access, ensuring equitability and maximizing prevention, early detection, follow-up and palliative care of cancer patients.

Without health data on structure, process and outcome, we cannot know if all programs and protocols that have been successful in wealthy settings will work as well in limited-resource settings [71]. This highlights the need for population-based effectiveness studies in developing countries [72]. Early evidence suggests that developed world protocols are not always as successful in developing world settings, probably due to a mix of patient-related, tumour-related, treatment-related and system-related factors [73-75]. Magrath et al found a higher rate of toxic deaths in their experience treating acute lymphoblastic leukemia in India compared to series from developed countries [73]. They hypothesized this was due to a combination of more extensive disease at diagnosis, delay in diagnosis, co-morbidity such as infection, differences in achievable home hygiene and poor access to acute and supportive care. The authors also noted large differences in regional leukemia genetics compared to Western series.

With limited resources, achieving the greatest health benefit with available resources is an important outcome of health systems. The vast majority of economic analyses for cancer treatment, palliation, screening and early detection are from developed countries [76]. Though there are clearly many cost-effective interventions that oncologists use in developed countries, their cost-effectiveness in specific resource-limited settings is usually not known [77]. As both cost and effectiveness of an intervention can be different in developing countries compared to developed countries, these must be measured and taken into account before determining whether an intervention would be cost-effective for a specific limited-resource country.

One example where an economic analysis has been done for developing countries is from the Breast Health Global Initiative (BHGI). BHGI produced regional estimates on the cost-effectiveness of treating breast cancer, comparing results for Africa, Asia and North America. The cost-effectiveness ratios for treating early-stage breast cancer versus not treating it were $78, $62 and $1960 per disability adjusted life-year (DALY) averted for the African, Asian and North American regions respectively [78]. A comprehensive breast cancer treatment program that treated early and advanced stages of breast cancer and involved early detection would cost $75, $75 and $915 per DALY averted in these respective regions.

Similarly, cost per fraction of radiation is highly sensitive to context. A single fraction of radiation has been estimated to cost $35 to deliver by a cobalt machine in the Netherlands versus $2 in China or India [79]. This relates to differences in the yearly number of fractions of radiation delivered per radiation machine, cost of quality assurance and machine maintenance as well as differences in capital cost of cobalt radiation machines. We would highlight the need for international cooperation and innovation to modify currently prohibitive radiation machine costs for developing countries.

Cost-effectiveness of cancer prevention and early detection in Low- and Middle-Income Countries has been assessed in some cases. Cervical cancer screening with visual inspection with acetic acid (VIA) with a one-visit twice-in-a-lifetime approach costs $91 per year of life saved in India and $319 per year of life saved in Kenya [80]. The authors place these figures in perspective by noting that a single lifetime screening with VIA is as cost-effective as hepatitis B vaccination in India and malaria prevention using bed nets in Kenya.

Methods and criteria for economic assessment of cancer treatments in low-resource settings are developing. As suggested by the Commission of Economics and Health, one approach is to assess the cost-appropriateness of a treatment by comparing the incremental cost per disability-adjusted life-year averted to the country's per capita gross domestic product (GDP) [76,81]. Using a similar method, Rawlins found that none of the 26 modern chemotherapy regimens assessed were cost-effective in 12 of 13 Low- and Middle-Income Asia-Pacific countries [82]. It is important to note these calculations assume drug costs are the same in developing countries as in the UK. Rawlins' findings emphasize the need for global action on appropriate cancer drug prices for developing countries.

The WHO-CHOICE program has also developed a method referred to as generalized cost-effectiveness analysis [83]. It allows for region-specific cost-effectiveness estimation. This can be of use to a developing country currently unable to afford the multiple economic analyses needed on numerous health interventions. The analysis of cost-effectiveness of breast cancer interventions by BHGI is one example of such an analysis. As another example, four interventions for tobacco control requiring government action were found cost-effective in most world regions using this methodology: taxation, clean indoor air laws, a comprehensive ban on advertising and information dissemination on the risks to health of tobacco [84]. Nicotine replacement also fell within the realm of cost-effective for many regions using the cut-off of cost per DALY averted of less than three times GDP per capita [84]. These modeled results do not eliminate the need for country-specific cost-effectiveness analyses but can be complimentary to them, helping inform decision making and sectoral debates on resource allocation [85].

### Health Data for Quality Cancer Control in Developing Countries: Summary

Greater investment in cancer control paired with the use of health data to guide and optimize investment holds the promise of improved cancer outcomes in developing countries. The benefits of health data for quality cancer control are summarized in table 1.

### Capacity for Health Services Research in Developing Countries

In addition to limitations in data availability, there is limited human capacity in Low- and Middle-Income Countries to produce health services and policy research [86]. Organizations such as AfrOx, the International Agency for Research on Cancer (IARC), the International Union Against Cancer (UICC) and others are supporting development of health services research capacity in developing countries. As mentioned previously, there has been a renewed commitment to improving health systems data in developing countries by many of the largest global health agencies [55].

Gonzalez Block and Mills suggest that 0.1% of total health care expenditure should be spent on health services and policy research in developing countries [86]. On average, current expenditure in developing countries is estimated to be 0.007% of total health care expenditure, 14 times below this recommended norm. There is a great need for ongoing national and international support to build health services research capacity in developing countries. The international health services and policy research community has much to offer in helping achieve this.

## Summary

In most developing countries, there are large knowledge gaps in the description of oncology structure, process and outcome. Where information is available, it demonstrates a picture of great need. Access to care and quality of care must be improved and economic efficiencies gained. Investment in cancer control for developing countries is desperately needed. We would recommend four linked priorities to optimize this investment: (1) National and regional capacity building in health services research, health policy and planning. (2) Informed investment in higher quality health data sources. (3) More economic evaluations relevant to oncology in developing countries. (4) Research into effective and cohesive models of cancer control appropriate to developing countries. Achieving these will require increased national, regional and international collaboration and political leadership. These four priorities are similar to goals for the global cardiovascular research community and reflect the call to action of eight of the largest global health agencies on health data [55,87]. We strongly support achievement of these priorities within the scope of health information systems and research agendas that cover the full scope of health conditions afflicting developing countries (horizontal integration). Health services research has an irreplaceable role in oncology for developing countries, equipping health systems to optimally and efficiently prevent the preventable, detect cancer as early as possible, cure the curable, and to relieve the intense suffering caused by cancer among those in need.

## Competing interests

The authors declare that they have no competing interests.

## Authors' contributions

TPH contributed to concept development, format, content, editing and writing of the manuscript. ACTK contributed to concept development, content and editing of the manuscript.

## Authors' Information

At the time of production of this article, TPH was a resident and clinical fellow in the Division of Radiation Oncology at the Cancer Centre of Southeastern Ontario. He was also a research fellow in oncology health services research at Queen’s University/ Queen’s Cancer Research Institute Division of Cancer Care and Epidemiology. ACTK is the Head of Epidemiology and Biostatistics at the Ocean Road Cancer Institute.

## Pre-publication history

The pre-publication history for this paper can be accessed here:

http://www.biomedcentral.com/1472-698X/10/24/prepub
